# Supplementary material for: Correlates of low birth weight and preterm birth in India
Source: PLoS One. 2023 Aug 17;18(8):e0287919. doi: 10.1371/journal.pone.0287919 (PMC10434923; doi:10.1371/journal.pone.0287919)
Supplement: Attachment — Submitted filename: Review.pdf [file pone.0287919.s003.pdf]

REPLY TO REVIEWERS' COMMENTS
Submission ID: PONE-D-22-27840
Title: Correlates of Low Birth Weight and Preterm Birth in India

I sincerely thank the editor and the anonymous reviewers for their appreciation, thoughtful suggestions and comments. I have revised the manuscript as per the suggestions and comments of the reviewers. A point-by-point reply to each of the reviewer's comment is given below.

Reviewer's comments:

Title: we note you've agreed with the reviewers and have now focussed your analysis on LBW and PTB. Based on this, we think your title can be simplified. e.g. "Correlates of Low Birth Weight and Pre-term Birth in India"

Response: Thank you for suggesting the title. I have modified the title as per your suggestion.

Abstract line 30: check use of word 'determinates'. This is a typo and should be corrected. Please do a thorough editorial review of the entire manuscript to ensure these issues are avoided.

Response: Thank you for the precise observation. I have revised the manuscript and removed all the typo mistakes.

Abstract lines 33-35: you're still maintaining the broader term 'adverse birth outcomes'- this should be corrected.

Response: Modified as per your suggestion.
