# Supplementary material for: Correlates of low birth weight and preterm birth in India
Source: PLoS One. 2023 Aug 17;18(8):e0287919. doi: 10.1371/journal.pone.0287919 (PMC10434923; doi:10.1371/journal.pone.0287919)
Supplement: Attachment — Submitted filename: Review Report-PLOS ONE.docx [file pone.0287919.s001.docx]

**Abstract**

The abstract is well-written, overall. However, the results section of the abstract requires some further clarification. For example, the author stated that “*However, a few correlates were found to be differently associated with PTB and LBW. Mothers who belonged to richer wealth status had a higher risk of PTB, the relationship was reversed in the case of LBW*” The authors fell short of reporting those few correlates. It would be instructive to report those different correlates.

**Introduction**

the current introduction section looks great, but I was expecting to see an overview of the prevalent adverse birth outcomes globally before the focus on the most common/prevalent ones. From the onset, the author explicitly identified PTB and LBW as the key adverse birth outcomes and missed the point about ensuring the readers have a sense of the others before narrowing on these two. If the author intends to proceed, then a title will need to be modified to reflect PTB and LBW. At least, the introduction section should provide the reader with sufficient information about the various adverse birth outcomes and some of the reported predictors.

Also, a see a bit of dissonance in reporting studies on general birth outcomes on one hand, and PTB and LBW on the other hand. I suggest a presentation of the literature first on adverse birth outcomes before a focus on the two most prevalent birth outcomes.

Is it possible to compare the state of the evidence on adverse birth outcomes globally and what pertains to India? Are there any differences or uniqueness between Indian and other countries?

**Methods and Results**

It will be important for the author to report the sampling approach adopted in the national survey from which the data was also sampled or extracted for this study. Also, was permission sought before the data was accessed or the data is freely accessible to the public? Again, it is clear from the methods and presentation of the results that the focus of the paper is PTB and LBW and so I suggest the title be modified to reflect this. I believe both bivariate and multivariable logistic regression analyses were carried out, so it is important to emphasize that.

Overall, the results as presented are okay but can I suggest the author reorganizes the results into two sections? The first can take the form of descriptive results where data on the prevalence and risk factors are presented. The second will be logistics regression which will present results including from models 1-4 as seen in table 2.

**Discussion**

There are some grammatical errors that need to be attended to via thorough proofreading. For example, see line 299. I believe the author meant to use the word “*off-track”.*
